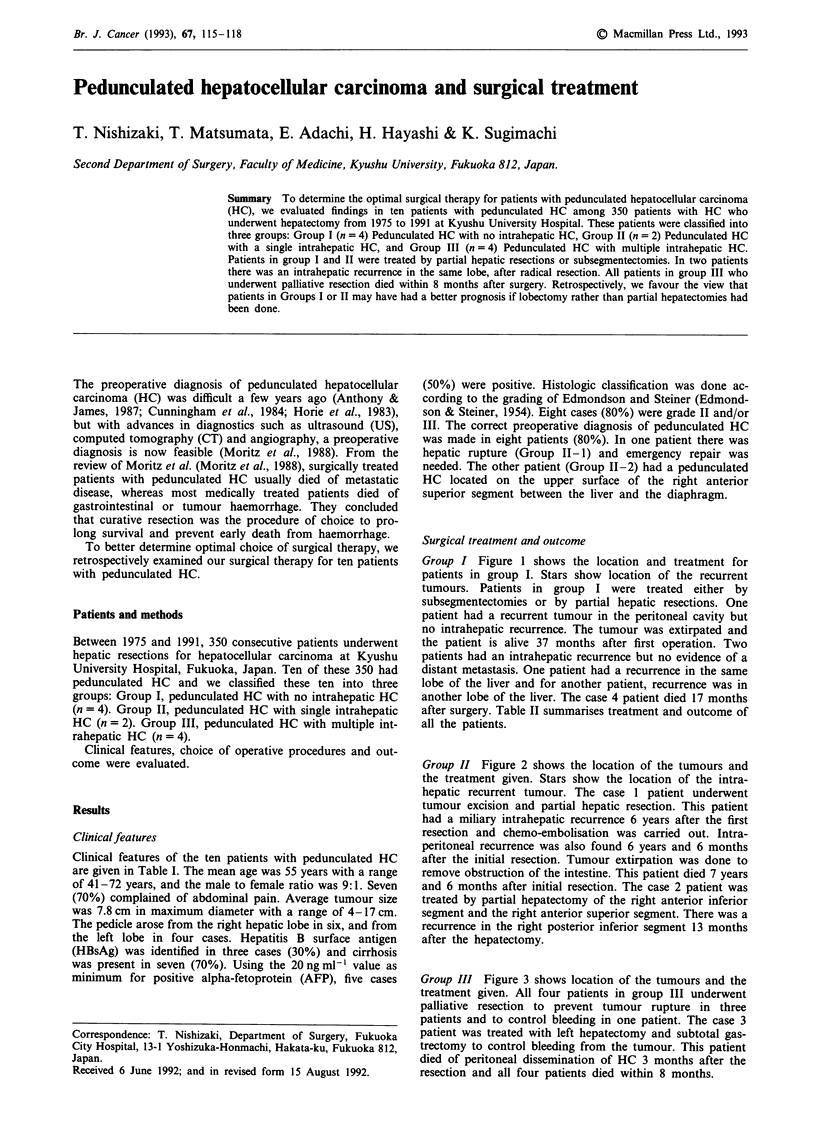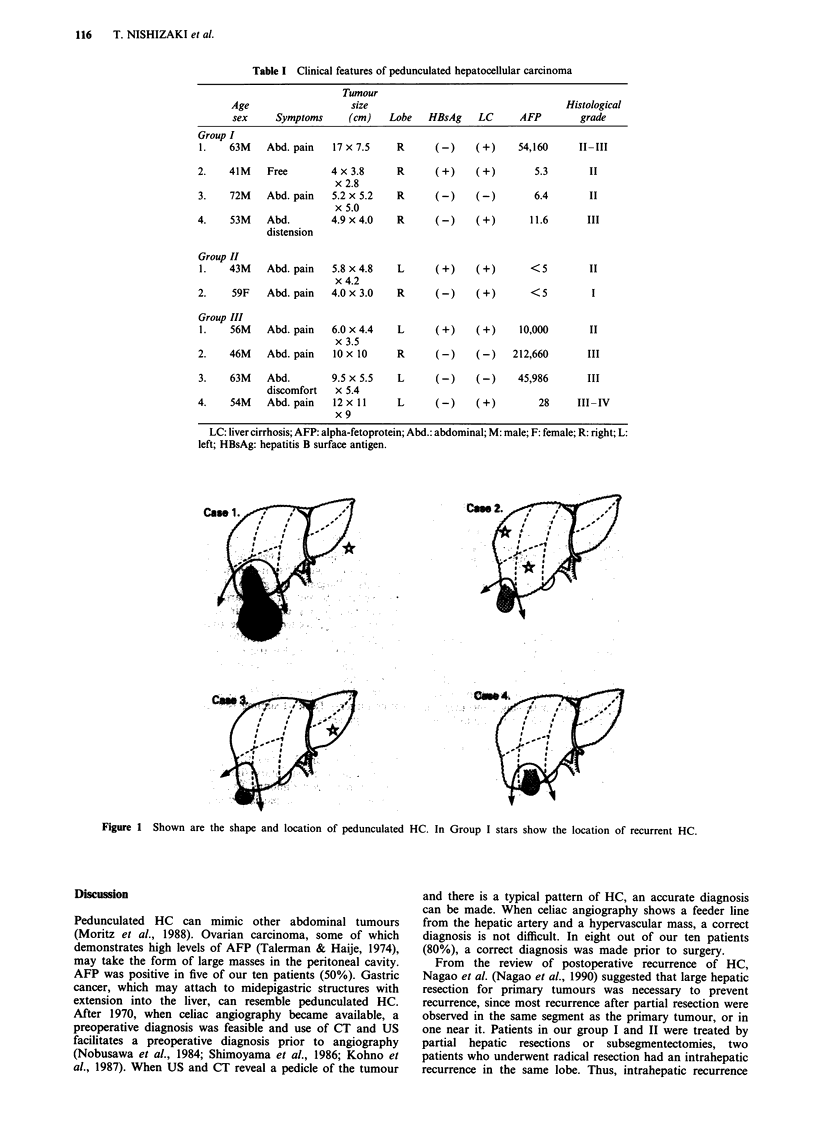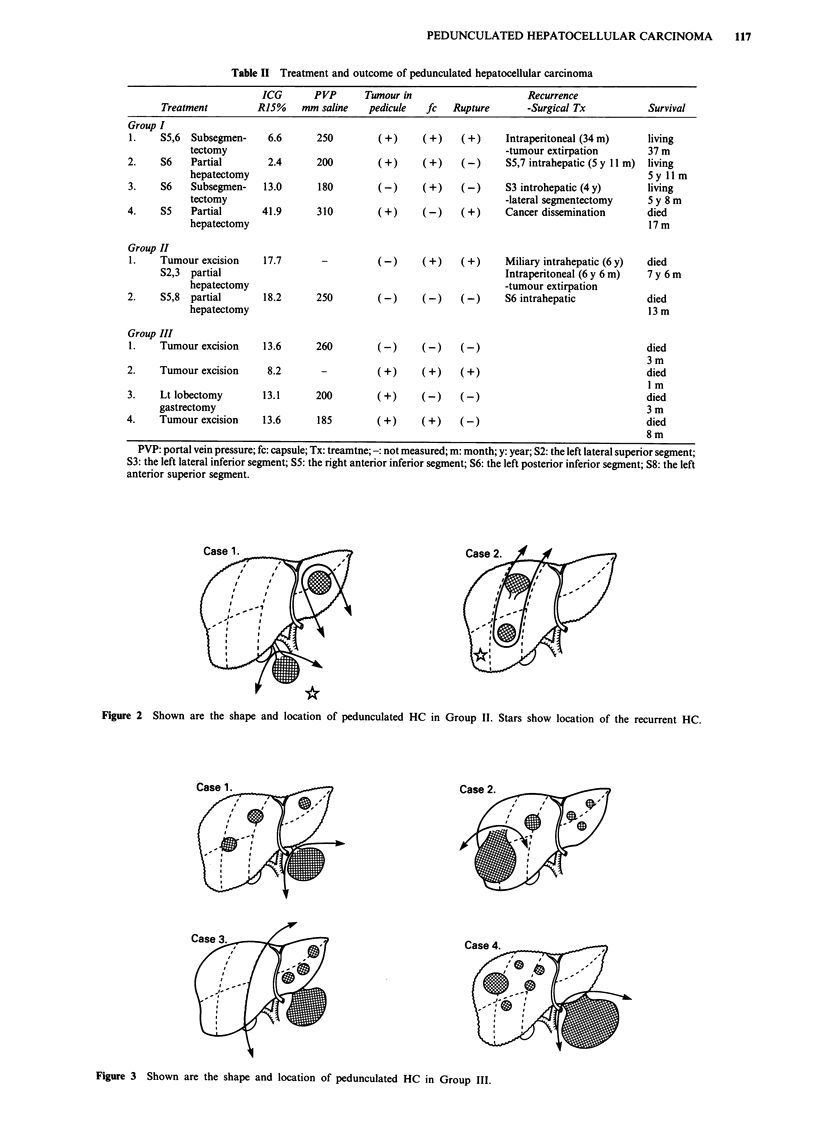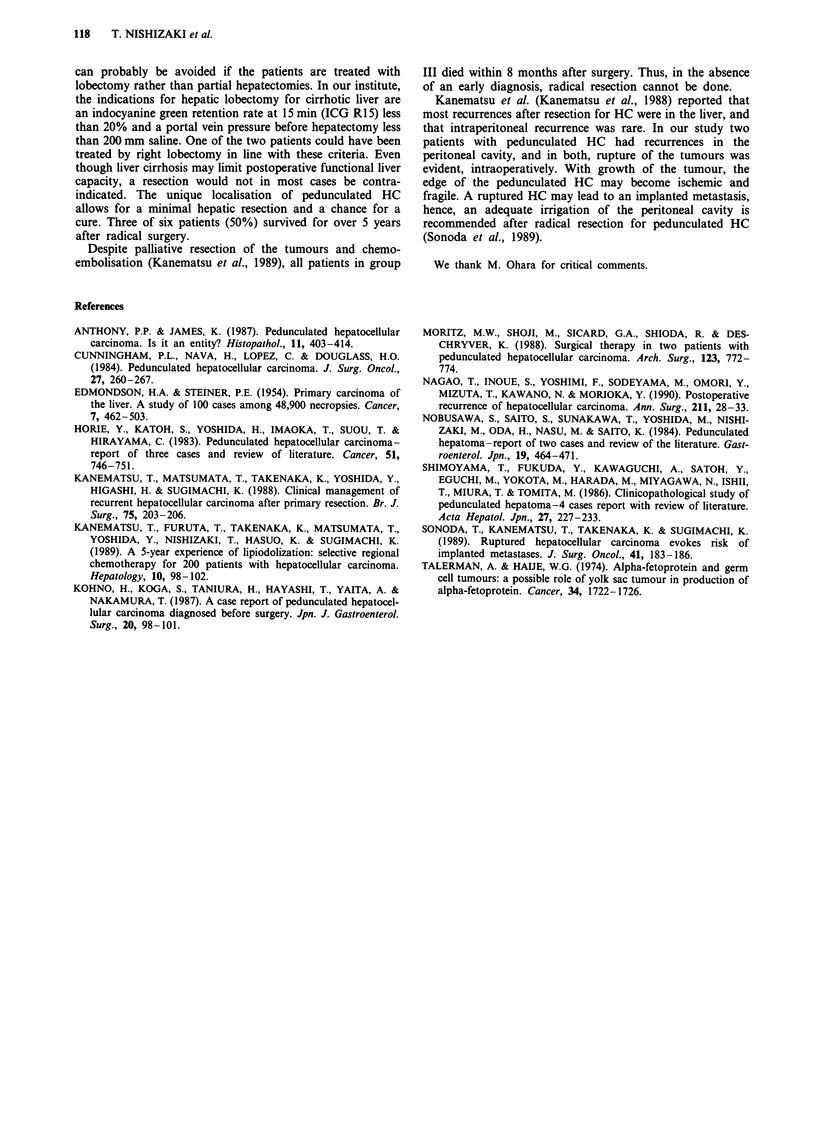# Pedunculated hepatocellular carcinoma and surgical treatment.

**DOI:** 10.1038/bjc.1993.20

**Published:** 1993-01

**Authors:** T. Nishizaki, T. Matsumata, E. Adachi, H. Hayashi, K. Sugimachi

**Affiliations:** Second Department of Surgery, Faculty of Medicine, Kyushu University, Fukuoka, Japan.

## Abstract

To determine the optimal surgical therapy for patients with pedunculated hepatocellular carcinoma (HC), we evaluated findings in ten patients with pedunculated HC among 350 patients with HC who underwent hepatectomy from 1975 to 1991 at Kyushu University Hospital. These patients were classified into three groups: Group I (n = 4) Pedunculated HC with no intrahepatic HC, Group II (n = 2) Pedunculated HC with a single intrahepatic HC, and Group III (n = 4) Pedunculated HC with multiple intrahepatic HC. Patients in group I and II were treated by partial hepatic resections or subsegmentectomies. In two patients there was an intrahepatic recurrence in the same lobe, after radical resection. All patients in group III who underwent palliative resection died within 8 months after surgery. Retrospectively, we favour the view that patients in Groups I or II may have had a better prognosis if lobectomy rather than partial hepatectomies had been done.


*Br. J. Cancer* (1993), **67**, 115–118 © Macmillan Press Ltd., 1993

### Pedunculated hepatocellular carcinoma and surgical treatment

T. Nishizaki, T. Matsumata, E. Adachi, H. Hayashi & K. Sugimachi

*Second Department of Surgery, Faculty of Medicine, Kyushu University, Fukuoka 812, Japan.*

**Summary** To determine the optimal surgical therapy for patients with pedunculated hepatocellular carcinoma (HC), we evaluated findings in ten patients with pedunculated HC among 350 patients with HC who underwent hepatectomy from 1975 to 1991 at Kyushu University Hospital. These patients were classified into three groups: Group I ($n = 4$) Pedunculated HC with no intrahepatic HC, Group II ($n = 2$) Pedunculated HC with a single intrahepatic HC, and Group III ($n = 4$) Pedunculated HC with multiple intrahepatic HC. Patients in group I and II were treated by partial hepatic resections or subsegmentectomies. In two patients there was an intrahepatic recurrence in the same lobe, after radical resection. All patients in group III who underwent palliative resection died within 8 months after surgery. Retrospectively, we favour the view that patients in Groups I or II may have had a better prognosis if lobectomy rather than partial hepatectomies had been done.

The preoperative diagnosis of pedunculated hepatocellular carcinoma (HC) was difficult a few years ago (Anthony & James, 1987; Cunningham *et al.*, 1984; Horie *et al.*, 1983), but with advances in diagnostics such as ultrasound (US), computed tomography (CT) and angiography, a preoperative diagnosis is now feasible (Moritz *et al.*, 1988). From the review of Moritz *et al.* (Moritz *et al.*, 1988), surgically treated patients with pedunculated HC usually died of metastatic disease, whereas most medically treated patients died of gastrointestinal or tumour haemorrhage. They concluded that curative resection was the procedure of choice to prolong survival and prevent early death from haemorrhage.

To better determine optimal choice of surgical therapy, we retrospectively examined our surgical therapy for ten patients with pedunculated HC.

### Patients and methods

Between 1975 and 1991, 350 consecutive patients underwent hepatic resections for hepatocellular carcinoma at Kyushu University Hospital, Fukuoka, Japan. Ten of these 350 had pedunculated HC and we classified these ten into three groups: Group I, pedunculated HC with no intrahepatic HC ($n = 4$). Group II, pedunculated HC with single intrahepatic HC ($n = 2$). Group III, pedunculated HC with multiple intrahepatic HC ($n = 4$).

Clinical features, choice of operative procedures and outcome were evaluated.

### Results

### *Clinical features*

Clinical features of the ten patients with pedunculated HC are given in Table I. The mean age was 55 years with a range of 41–72 years, and the male to female ratio was 9:1. Seven (70%) complained of abdominal pain. Average tumour size was 7.8 cm in maximum diameter with a range of 4–17 cm. The pedicle arose from the right hepatic lobe in six, and from the left lobe in four cases. Hepatitis B surface antigen (HBsAg) was identified in three cases (30%) and cirrhosis was present in seven (70%). Using the 20 ng ml$^{-1}$ value as minimum for positive alpha-fetoprotein (AFP), five cases (50%) were positive. Histologic classification was done according to the grading of Edmondson and Steiner (Edmondson & Steiner, 1954). Eight cases (80%) were grade II and/or III. The correct preoperative diagnosis of pedunculated HC was made in eight patients (80%). In one patient there was hepatic rupture (Group II–1) and emergency repair was needed. The other patient (Group II–2) had a pedunculated HC located on the upper surface of the right anterior superior segment between the liver and the diaphragm.

### *Surgical treatment and outcome*

*Group I* Figure 1 shows the location and treatment for patients in group I. Stars show location of the recurrent tumours. Patients in group I were treated either by subsegmentectomies or by partial hepatic resections. One patient had a recurrent tumour in the peritoneal cavity but no intrahepatic recurrence. The tumour was extirpated and the patient is alive 37 months after first operation. Two patients had an intrahepatic recurrence but no evidence of a distant metastasis. One patient had a recurrence in the same lobe of the liver and for another patient, recurrence was in another lobe of the liver. The case 4 patient died 17 months after surgery. Table II summarises treatment and outcome of all the patients.

*Group II* Figure 2 shows the location of the tumours and the treatment given. Stars show the location of the intrahepatic recurrent tumour. The case 1 patient underwent tumour excision and partial hepatic resection. This patient had a miliary intrahepatic recurrence 6 years after the first resection and chemo-embolisation was carried out. Intraperitoneal recurrence was also found 6 years and 6 months after the initial resection. Tumour extirpation was done to remove obstruction of the intestine. This patient died 7 years and 6 months after initial resection. The case 2 patient was treated by partial hepatectomy of the right anterior inferior segment and the right anterior superior segment. There was a recurrence in the right posterior inferior segment 13 months after the hepatectomy.

*Group III* Figure 3 shows location of the tumours and the treatment given. All four patients in group III underwent palliative resection to prevent tumour rupture in three patients and to control bleeding in one patient. The case 3 patient was treated with left hepatectomy and subtotal gastrectomy to control bleeding from the tumour. This patient died of peritoneal dissemination of HC 3 months after the resection and all four patients died within 8 months.

Correspondence: T. Nishizaki, Department of Surgery, Fukuoka City Hospital, 13-1 Yoshizuka-Honmachi, Hakata-ku, Fukuoka 812, Japan.
Received 6 June 1992; and in revised form 15 August 1992.

**Table I** Clinical features of pedunculated hepatocellular carcinoma

| | *Age sex* | *Symptoms* | *Tumour size (cm)* | *Lobe* | *HBsAg* | *LC* | *AFP* | *Histological grade* |
|---|---|---|---|---|---|---|---|---|
| *Group I* | | | | | | | | |
| 1. | 63M | Abd. pain | 17 × 7.5 | R | (−) | (+) | 54,160 | II–III |
| 2. | 41M | Free | 4 × 3.8 × 2.8 | R | (+) | (+) | 5.3 | II |
| 3. | 72M | Abd. pain | 5.2 × 5.2 × 5.0 | R | (−) | (−) | 6.4 | II |
| 4. | 53M | Abd. distension | 4.9 × 4.0 | R | (−) | (+) | 11.6 | III |
| *Group II* | | | | | | | | |
| 1. | 43M | Abd. pain | 5.8 × 4.8 × 4.2 | L | (+) | (+) | <5 | II |
| 2. | 59F | Abd. pain | 4.0 × 3.0 | R | (−) | (+) | <5 | I |
| *Group III* | | | | | | | | |
| 1. | 56M | Abd. pain | 6.0 × 4.4 × 3.5 | L | (+) | (+) | 10,000 | II |
| 2. | 46M | Abd. pain | 10 × 10 | R | (−) | (−) | 212,660 | III |
| 3. | 63M | Abd. discomfort | 9.5 × 5.5 × 5.4 | L | (−) | (−) | 45,986 | III |
| 4. | 54M | Abd. pain | 12 × 11 × 9 | L | (−) | (+) | 28 | III–IV |

LC: liver cirrhosis; AFP: alpha-fetoprotein; Abd.: abdominal; M: male; F: female; R: right; L: left; HBsAg: hepatitis B surface antigen.

**Figure 1** Shown are the shape and location of pedunculated HC. In Group I stars show the location of recurrent HC.

### Discussion

Pedunculated HC can mimic other abdominal tumours (Moritz *et al.*, 1988). Ovarian carcinoma, some of which demonstrates high levels of AFP (Talerman & Haije, 1974), may take the form of large masses in the peritoneal cavity. AFP was positive in five of our ten patients (50%). Gastric cancer, which may attach to midepigastric structures with extension into the liver, can resemble pedunculated HC. After 1970, when celiac angiography became available, a preoperative diagnosis was feasible and use of CT and US facilitates a preoperative diagnosis prior to angiography (Nobusawa *et al.*, 1984; Shimoyama *et al.*, 1986; Kohno *et al.*, 1987). When US and CT reveal a pedicle of the tumour and there is a typical pattern of HC, an accurate diagnosis can be made. When celiac angiography shows a feeder line from the hepatic artery and a hypervascular mass, a correct diagnosis is not difficult. In eight out of our ten patients (80%), a correct diagnosis was made prior to surgery.

From the review of postoperative recurrence of HC, Nagao *et al.* (Nagao *et al.*, 1990) suggested that large hepatic resection for primary tumours was necessary to prevent recurrence, since most recurrence after partial resection were observed in the same segment as the primary tumour, or in one near it. Patients in our group I and II were treated by partial hepatic resections or subsegmentectomies, two patients who underwent radical resection had an intrahepatic recurrence in the same lobe. Thus, intrahepatic recurrence

**Table II** Treatment and outcome of pedunculated hepatocellular carcinoma

| | Treatment | ICG R15% | PVP mm saline | Tumour in pedicule | fc | Rupture | Recurrence -Surgical Tx | Survival |
|---|---|---|---|---|---|---|---|---|
| *Group I* | | | | | | | | |
| 1. | S5,6 Subsegmentectomy | 6.6 | 250 | (+) | (+) | (+) | Intraperitoneal (34 m) -tumour extirpation | living 37 m |
| 2. | S6 Partial hepatectomy | 2.4 | 200 | (+) | (+) | (−) | S5,7 intrahepatic (5 y 11 m) | living 5 y 11 m |
| 3. | S6 Subsegmentectomy | 13.0 | 180 | (−) | (+) | (−) | S3 introhepatic (4 y) -lateral segmentectomy | living 5 y 8 m |
| 4. | S5 Partial hepatectomy | 41.9 | 310 | (+) | (−) | (+) | Cancer dissemination | died 17 m |
| *Group II* | | | | | | | | |
| 1. | Tumour excision S2,3 partial hepatectomy | 17.7 | – | (−) | (+) | (+) | Miliary intrahepatic (6 y) Intraperitoneal (6 y 6 m) -tumour extirpation | died 7 y 6 m |
| 2. | S5,8 partial hepatectomy | 18.2 | 250 | (−) | (−) | (−) | S6 intrahepatic | died 13 m |
| *Group III* | | | | | | | | |
| 1. | Tumour excision | 13.6 | 260 | (−) | (−) | (−) | | died 3 m |
| 2. | Tumour excision | 8.2 | – | (+) | (+) | (+) | | died 1 m |
| 3. | Lt lobectomy gastrectomy | 13.1 | 200 | (+) | (−) | (−) | | died 3 m |
| 4. | Tumour excision | 13.6 | 185 | (+) | (+) | (−) | | died 8 m |

PVP: portal vein pressure; fc: capsule; Tx: treamtne; –: not measured; m: month; y: year; S2: the left lateral superior segment; S3: the left lateral inferior segment; S5: the right anterior inferior segment; S6: the left posterior inferior segment; S8: the left anterior superior segment.

**Figure 2** Shown are the shape and location of pedunculated HC in Group II. Stars show location of the recurrent HC.

**Figure 3** Shown are the shape and location of pedunculated HC in Group III.

can probably be avoided if the patients are treated with lobectomy rather than partial hepatectomies. In our institute, the indications for hepatic lobectomy for cirrhotic liver are an indocyanine green retention rate at 15 min (ICG R15) less than 20% and a portal vein pressure before hepatectomy less than 200 mm saline. One of the two patients could have been treated by right lobectomy in line with these criteria. Even though liver cirrhosis may limit postoperative functional liver capacity, a resection would not in most cases be contraindicated. The unique localisation of pedunculated HC allows for a minimal hepatic resection and a chance for a cure. Three of six patients (50%) survived for over 5 years after radical surgery.

Despite palliative resection of the tumours and chemoembolisation (Kanematsu *et al.*, 1989), all patients in group III died within 8 months after surgery. Thus, in the absence of an early diagnosis, radical resection cannot be done.

Kanematsu *et al.* (Kanematsu *et al.*, 1988) reported that most recurrences after resection for HC were in the liver, and that intraperitoneal recurrence was rare. In our study two patients with pedunculated HC had recurrences in the peritoneal cavity, and in both, rupture of the tumours was evident, intraoperatively. With growth of the tumour, the edge of the pedunculated HC may become ischemic and fragile. A ruptured HC may lead to an implanted metastasis, hence, an adequate irrigation of the peritoneal cavity is recommended after radical resection for pedunculated HC (Sonoda *et al.*, 1989).

We thank M. Ohara for critical comments.

### References

ANTHONY, P.P. & JAMES, K. (1987). Pedunculated hepatocellular carcinoma. Is it an entity? *Histopathol.*, **11**, 403–414.

CUNNINGHAM, P.L., NAVA, H., LOPEZ, C. & DOUGLASS, H.O. (1984). Pedunculated hepatocellular carcinoma. *J. Surg. Oncol.*, **27**, 260–267.

EDMONDSON, H.A. & STEINER, P.E. (1954). Primary carcinoma of the liver. A study of 100 cases among 48,900 necropsies. *Cancer*, **7**, 462–503.

HORIE, Y., KATOH, S., YOSHIDA, H., IMAOKA, T., SUOU, T. & HIRAYAMA, C. (1983). Pedunculated hepatocellular carcinoma-report of three cases and review of literature. *Cancer*, **51**, 746–751.

KANEMATSU, T., MATSUMATA, T., TAKENAKA, K., YOSHIDA, Y., HIGASHI, H. & SUGIMACHI, K. (1988). Clinical management of recurrent hepatocellular carcinoma after primary resection. *Br. J. Surg.*, **75**, 203–206.

KANEMATSU, T., FURUTA, T., TAKENAKA, K., MATSUMATA, T., YOSHIDA, Y., NISHIZAKI, T., HASUO, K. & SUGIMACHI, K. (1989). A 5-year experience of lipiodolization: selective regional chemotherapy for 200 patients with hepatocellular carcinoma. *Hepatology*, **10**, 98–102.

KOHNO, H., KOGA, S., TANIURA, H., HAYASHI, T., YAITA, A. & NAKAMURA, T. (1987). A case report of pedunculated hepatocellular carcinoma diagnosed before surgery. *Jpn. J. Gastroenterol. Surg.*, **20**, 98–101.

MORITZ, M.W., SHOJI, M., SICARD, G.A., SHIODA, R. & DESCHRYVER, K. (1988). Surgical therapy in two patients with pedunculated hepatocellular carcinoma. *Arch. Surg.*, **123**, 772–774.

NAGAO, T., INOUE, S., YOSHIMI, F., SODEYAMA, M., OMORI, Y., MIZUTA, T., KAWANO, N. & MORIOKA, Y. (1990). Postoperative recurrence of hepatocellular carcinoma. *Ann. Surg.*, **211**, 28–33.

NOBUSAWA, S., SAITO, S., SUNAKAWA, T., YOSHIDA, M., NISHIZAKI, M., ODA, H., NASU, M. & SAITO, K. (1984). Pedunculated hepatoma-report of two cases and review of the literature. *Gastroenterol. Jpn.*, **19**, 464–471.

SHIMOYAMA, T., FUKUDA, Y., KAWAGUCHI, A., SATOH, Y., EGUCHI, M., YOKOTA, M., HARADA, M., MIYAGAWA, N., ISHII, T., MIURA, T. & TOMITA, M. (1986). Clinicopathological study of pedunculated hepatoma-4 cases report with review of literature. *Acta Hepatol. Jpn.*, **27**, 227–233.

SONODA, T., KANEMATSU, T., TAKENAKA, K. & SUGIMACHI, K. (1989). Ruptured hepatocellular carcinoma evokes risk of implanted metastases. *J. Surg. Oncol.*, **41**, 183–186.

TALERMAN, A. & HAIJE, W.G. (1974). Alpha-fetoprotein and germ cell tumours: a possible role of yolk sac tumour in production of alpha-fetoprotein. *Cancer*, **34**, 1722–1726.